# “We need to accept our limited resources”: a qualitative study exploring ambulance clinicians’ experiences of working conditions when caring for patients with breathlessness

**DOI:** 10.1186/s12873-026-01620-w

**Published:** 2026-05-28

**Authors:** Wivica Kauppi, Karl Hedman, Hanna Maurin Söderholm, Magnus Andersson Hagiwara

**Affiliations:** 1 PreHospen- Centre for Prehospital Research, Faculty of Caring Science, Work Life and Social Welfare, University of Borås, Borås, Sweden; 2 Faculty of Caring Science, Work Life and Social Welfare, University of Borås, Borås, Sweden; 3 PICTA- Prehospital Innovation Arena, Lindholmen Science Park, Gothenburg, Sweden

**Keywords:** Breathlessness, Dyspnoea, Ambulance services, Emergency medical services, Ambulance clinician, Work condition, Environment, Caring, Safety

## Abstract

**Background:**

Breathlessness is a common and potentially life-threatening symptom among patients and constitutes a frequent reason for ambulance dispatches. Within ambulance care, ambulance clinicians (ACs) are often required to make rapid clinical assessments and decisions under complex and unpredictable conditions. Despite this, there is limited knowledge of how ACs experience their working conditions in ambulance care when caring for patients with breathlessness. Little is known about how these working conditions influence their ability to balance clinical responsibility, patient needs, and environmental constraints in the ambulance care setting. This study aimed to explore how ACs experience their working conditions in connection with the care of patients with breathlessness.

**Methods:**

This qualitative exploratory sub-study included a sample of 16 ACs from southwestern Sweden, all with at least one year of clinical experience. Data were collected through semi-structured dyadic interviews following a simulation exercise and analyzed using reflexive thematic analysis.

**Results:**

The ACs experiences of their working conditions in connection with the care of patients with breathlessness is captured in three main higher-order themes: navigating constraints of the care environment, navigating professional judgment within institutional boundaries, and navigating responsibility in uncertain and distant care situations. ACs highlighted situations in which high-acuity care is delivered in environments that do not consistently ensure clinician safety. Clinical guidelines support decision-making in high-stress situations but may become restrictive when patients’ conditions fall outside standard recommendations or when interventions are contraindicated. To address these constraints, ACs develop adaptive strategies. Non-conveyance decisions involving patients with breathlessness are described as particularly complex and burdensome, a challenge intensified by a shift toward selective transport and increased reliance on individual clinical judgment under uncertainty.

**Conclusion:**

The findings show that working conditions in ambulance services influence how care for patients with breathlessness is provided and experienced. Care is delivered in contexts marked by urgency, uncertainty, and constrained resources, requiring ACs to navigate patient needs, safety considerations, and organizational expectations. While acceptance of limitations functions as a professional strategy, it carries ethical implications for patients’ safety, dignity, and care.

**Supplementary Information:**

The online version contains supplementary material available at 10.1186/s12873-026-01620-w.

## Background

Patients experiencing breathlessness (dyspnoea) is one of the most common reasons for ambulance activation worldwide [1]. Breathlessness refers to a subjective experience of breathing discomfort expressed through sensations such as greater exertion required to breathe, a feeling of inadequate air, or chest constriction [2]. It can arise from a variety of underlying causes, including chronic or episodic conditions such as anxiety, pneumonia, chronic obstructive pulmonary disease, and asthma. It may also be a symptom of acute, potentially life-threatening events, such as acute heart failure, pulmonary embolism, or myocardial infarction [1, 3, 4], and is associated with high mortality rate [1, 5, 6].

Pre-hospital organizations, including ambulance services, differ significantly across countries [7]. These structural variations influence how ambulance clinicians (ACs) carry out their work, including differences in response systems, staffing models, and available resources. Despite these differences, ambulance services share a common mission, to deliver timely and safe care in situations where patient needs and circumstances vary widely [8]. In Sweden, ambulance services constitute an integral part of the national healthcare system and are governed by legislation that ensures equal and high-quality care for all citizens [9]. Operational conditions differ across regions depending on population density, geography, and local infrastructure [10]. The Swedish National regulations require that each ambulance is staffed with at least one registered nurse (RN). A majority of ACs have postgraduate education in pre-hospital emergency care, although some have additional training in areas such as intensive care or anesthesia. Ambulance crews in Sweden typically consist of two personnel: an AC and an emergency medical technician, who functions as an assistant nurse. No dedicated driver is included, as both crew members share responsibility for patient care and vehicle operation. Physicians are mainly involved in advisory functions or within specialized units such as helicopter emergency medical services [11].

ACs work around the clock in settings where they must make decisions independently and without direct access to hospital resources [12, 13]. Their practice is guided by medical guidelines, triage systems, and local protocols that structure assessment, treatment, and decisions regarding transport to an appropriate level of care. In certain situations, additional support may be provided through collaboration with, for example, the police, fire and rescue services, or other healthcare professionals across different levels of care [14, 15]. In caring for patients with breathlessness, specific demands are placed on ACs to provide personalized care from a holistic perspective, requiring attention not only to physical symptoms but also to psychological distress, existential concerns, and social needs that may arise in the ambulance care context [16, 17]. These patients often require rapid and repeated assessment, close monitoring, and supportive communication, as breathlessness may generate both physiological instability and considerable emotional distress [18, 19]. Previous research also shows that patients implicitly expect ACs to provide the best possible care and to possess the competence required to meet their needs, regardless of the severity of their condition [19]. In the ambulance setting, caring for patients with breathlessness can be particularly challenging, especially in time-critical situations [20]. Because breathlessness presents varying symptoms and degrees of severity, ACs are expected to quickly identify underlying causes through structured assessment and careful interpretation of symptoms. Early recognition of warning signs is essential to guide appropriate care provision and ensure patient safety [21, 22]. These expectations contribute to demanding working conditions for ACs, characterized by high levels of responsibility, rapid decision-making under uncertainty, and sustained emotional and cognitive strain when caring for vulnerable patients in time-critical and high-acuity situations [20]. Other factors that may contribute to challenging working conditions include clinical guidelines that are not well adapted to the pre-hospital context [13], as well as sometimes long transport distances with critically ill patients [23].

The Swedish ambulance services, like other countries, has become increasingly complex in recent years, with higher call volumes, growing expectations for advanced on-scene care, and an expansion of non-conveyance responsibilities [24]. This development places greater demands on ACs, requiring more extensive medical assessment and on-scene risk evaluation [25–27]. A recent result on ACs’ experiences of caring for patients with breathlessness shows several challenges in responding to patients’ individual needs, particularly in critical situations. Likewise, the findings indicate that ACs’ own vulnerability affected their ability to provide care, which created challenges in being genuinely present and in meeting patients’ needs for support [20]. Despite the high prevalence of breathlessness-related calls, limited research has explored how ACs experience working conditions, particularly regarding the interplay between clinical responsibilities, patient needs, and work environment challenges. Against this background, the aim of the study was to explore how ACs experience their working conditions in connection with the care of patients with breathlessness.

## Methods

### Study design

This study represents a secondary analysis of a previously collected qualitative dataset. The initial study used qualitative content analysis to describe ACs´ experiences of caring for patients with breathlessness [20]. Although the original aim focused on care experiences, the open-ended interviews also generated rich and unanticipated data on working conditions that were not fully explored in the primary analysis. These data were considered analytically meaningful and form the basis of the present study. The same participants and overall procedures were retained, using a qualitative design with an inductive approach. This approach allowed patterns and themes to emerge directly from the participants’ experiences, rather than being constrained by predefined theoretical frameworks [28]. In contrast, the present study employs reflexive thematic analysis [29] to enable a more interpretive level of analysis, with a focus on themes, patterns of shared meanings, meaning construction, and how working conditions shape and influence experiences.

To provide a shared point of departure for reflection, data generation began with a simulated ambulance scenario created to resemble a realistic clinical situation. The simulation itself was not subject to analysis, instead it served as a reflective stimulus during the subsequent dyadic interviews. In these interviews, two ACs at a time discussed their thoughts and experiences, drawing on both the simulated event and their previous encounters with patients experiencing breathlessness. The integration of simulation and dyadic interviewing facilitated rich, experience-based discussions and contributed to a nuanced understanding of caregiving in the context of ambulance services [30, 31]. Data collection was carried out over two consecutive days in late May and early June 2021. Reporting of the study adhered to the COREQ (Consolidated Criteria for Reporting Qualitative Research) checklist to ensure transparency and completeness [32] (see Supplementary file 1).

### Participants and settings

The study was conducted in two ambulance organizations in southwestern Sweden, comprising five ambulance stations serving urban, suburban, and rural populations. Participant recruitment began with purposive sampling and was later complemented by snowball sampling due to the difficulty of reaching ACs who were willing to participate on the scheduled data collection days and during off-duty hours [33]. Inclusion criteria required participants to be RNs working in ambulance services with specialist training in ambulance care, intensive care, or anesthesia. Efforts were made to ensure variation in age, gender, clinical experience, and representation across urban, suburban, and rural service areas, as well as across the participating ambulance stations.

Following approval from chief executive officers, recruitment was conducted through internal communication platforms, social media postings, and targeted invitations. Participants who consented to take part were able to choose between two data collection days, and preferences regarding collaboration partners during the simulation and interview were accommodated when possible. No participants withdrew. A total of 16 ACs participated. The sample represented a broad range of ambulance stations and service environments, contributing to diverse perspectives. Participant characteristics are presented in Table 1, which is reused from our previous publication involving the same sample [20].

**Table 1:** Participants characteristics

	*n* = 16
**Gender**	
Male	11
Female	5
**Age (years)**	
25–34	5
35–44	5
45–54	6
**Clinical experience in ambulance services (years)**	
0–3 years	3
4–6 years	6
7–10 years	2
11–15 years	2
16–22 years	3
**Registered nurse with area of specialization**	
Ambulance care	12
Intensive care	1
Anesthesia care	1
Ambulance- and Intensive care	1
Anesthesia- and Intensive care	1

### The simulation

Before the interviews, participants worked in pairs as ambulance crew members in a standardized simulation involving a breathless patient scenario conducted in a fully equipped ambulance. A single trained actor portrayed the patient across all simulations to ensure consistency [34]. Participants were informed about the study’s general aims and instructed to act as they normally would [35]. Further details on the simulation procedure are provided in sub-study 1 [20].

### Data collection

Data was collected through eight dyadic interviews, in which two participants were interviewed together [36]. Following the simulations, semi-structured interviews were conducted face-to-face near the simulation site, allowing for immediate and contextually rich insights into participants’ experiences. Participants reflected on the simulation and related it to their prior experiences caring for patients with breathlessness in ambulance services. The interview guide was partly informed by previous studies on patients’ experiences of breathlessness during ambulance care [18, 19], but questions were kept open to allow an inductive exploration of participants’ own perspectives. Although designed to explore care experiences, the guide generated rich, unanticipated data on working conditions, which were not explicitly prompted but enabled a secondary analysis aligned with the present aim. Each interview began with the question, “How did you experience caring for this patient who was struggling with her breathing?” (see Supplementary file 2 for additional questions, reused from our previous publication [20]). Follow-up prompts encouraged elaboration, such as “What do you mean by…?”, “Can you describe it more?”, and “Can you give an example of…?”. All interviews were conducted in Swedish and transcribed verbatim in the original language. Data analysis was performed in Swedish to preserve meaning and contextual nuance. For manuscript preparation, selected quotes and the final manuscript were translated into English by the first author and reviewed for accuracy by the second author. Interviews lasted between 23 and 47 min, with a median of 39 min. They were conducted by the first author (*n* = 5), the third author (*n* = 1), and an external researcher experienced in qualitative interviews (*n* = 2). This distribution accommodated scheduling constraints and the first author’s prior connections with three of the participating ambulance crews. Data collection was not intended to achieve saturation. Instead, it included participants who expressed interest in taking part in the simulation and interview. Despite this, the interviews generated rich material that was sufficient to address the study aim. All interviews were audio-recorded, transcribed verbatim, and securely stored with access restricted to the study authors.

### Ethical considerations

The study received approval from the Swedish Ethical Review Authority (Dnr 2019–03283) and was conducted in accordance with the ethical principles of the Helsinki Declaration [37]. Participants received both written and verbal information about the study’s purpose, design, and their right to withdraw at any time without any consequences. Informed consent was obtained from all participants prior to data collection. All procedures, including data handling and data storage, were carried out in compliance with the General Data Protection Regulation (GDPR) and the university’s data management policies.

### Data analysis

The interviews were analyzed using the reflexive thematic analysis process described by Braun and Clarke [28, 38, 39]. Reflexivity acknowledges the active role of the researcher in the production of knowledge, recognizing how the researcher influences the meaning-making process and the resulting themes. This process is shaped by the dataset, including how the interviews were conducted, the theoretical assumptions brought to the analysis, and the researcher’s analytical skills and resources [38]. To structure and systematize the interview data, Braun and Clarke´s six-phase process was applied [28, 40], with elaborations suggested by Byrne [41]. First, the data were thoroughly reviewed to gain familiarity with their content. Second, interesting features across the dataset were systematically coded. Third, codes were organized into potential themes, and all relevant data were collated for each theme. Fourth, themes were evaluated to determine whether they aligned with coded extracts and the dataset. In the fifth step, themes were further refined, capturing shared meanings and developing clear definitions and labels for each theme. Finally, illustrative examples were selected, and the analysis was related to the research question, relevant theory, and previous findings. Steps one through four were conducted independently by the first and second author, followed by collaborative comparison and refinement of thematic patterns in steps five and six, including renaming and adjustments based on collective discussion of the individually generated themes.

### Trustworthiness

To enhance trustworthiness of the study [41, 42], all authors engaged in reflection on their pre-understandings and potential biases. The first and second authors conducted the analysis with care, with the first author performing the first coding and organizing the data into preliminary themes, while the second author critically reviewed these steps. This iterative process involved continuously challenging interpretations, identifying potential blind spots, and fostering deeper reflexivity, ensuring that the emerging themes were firmly grounded in the participants’ perspectives. Constructive input from the last author was incorporated throughout the revision process, enhancing transparency and clarity. As the dataset was extensive, it was analyzed through separate but complementary sub-studies guided by different analytical aims. This approach enabled an in-depth exploration of distinct aspects of the same empirical material while maintaining methodological coherence and transparency. To further strengthen confirmability, illustrative quotations from the interviews were included in the presentation of findings, allowing readers to trace the analysis back to the original data.

## Results

**Fig. 1:**
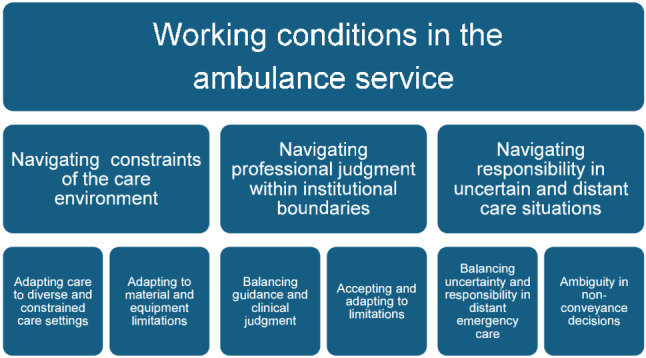
Thematic structure illustrating working conditions in the ambulance service

The results are organized under the overarching theme “Working conditions in the ambulance service”, which encompasses three main higher-order themes (Fig. 1): navigating constraints of the care environment, navigating professional judgment within institutional boundaries, and navigating responsibility in uncertain and distant care situations.

### Working conditions in the ambulance service

Working conditions profoundly shape ACs’ experiences in caring for patients with breathlessness in ambulance care. This overarching theme encompasses the physical, material, and organizational constraints of the ambulance environment, where limited vehicle space, equipment quality, and resource availability demand constant adaptation to ensure patient safety and effective care. ACs navigate institutional guidelines and professional judgment, balancing protocol adherence with flexible decision-making amid treatment limitations, while accepting the boundaries of ambulance care capabilities to prioritize stabilization during transport.

### Navigating constraints of the care environment

#### Adapting care to diverse and constrained care settings

In caring for patients with breathlessness, the physical environment in which ACs provide care significantly shapes their ability to act. ACs described how the design and size of the vehicle affect both patient care and their own working conditions. In larger ambulances, there is sufficient space for two ACs to work safely and effectively, whereas smaller vehicles restrict movement and make it difficult to deliver care without compromising patient safety.

*One thing that matters is the vehicle we have. When we have these patients*,* this vehicle is quite large*,* making it easy to move around and for two people to care for them in the back. We have other vehicles where it’s not even possible to care for two people safely*,* and it’s quite cramped*,* so that can make a difference. (Int8/Part1)*

ACs emphasize that these spatial limitations also affect their own safety. For instance, in situations where patients’ conditions are considered critical, ACs are sometimes in the ambulance forced to stand unrestrained to secure the airway of the patients or perform urgent emergency medical treatments. Such conditions are described as challenging and potentially unsafe yet accepted as part of the pre-hospital ambulance context.

*From a caregiver’s perspective*,* if things go wrong here*,* I might not be able to maintain clear airways*,* or I might have to stand without a seatbelt and stand up in the vehicle. (Int8/Part1).*

ACs highlight that the confined environment influences patients´ sense of safety and well-being. They reflect that small, enclosed vehicles can increase patients’ discomfort or anxiety, as the limited space forces proximity to equipment and ACs themselves.

*I believe that sometimes patients can feel uncomfortable when we’re in the Volvo*,* which is quite cramped. There are a lot of cables*,* and we often must reach over the patient… This situation can increase their stress*,* as they might feel surrounded by equipment and objects placed on them due to limited space (Int8/Part1).*

Beyond the ambulance, other confined environments, such as narrow apartments or steep staircases, are described by ACs as physically demanding and logistically complex. These situations are particularly challenging when caring for patients suffering from severe breathlessness. ACs emphasize that such circumstances require both creativity and teamwork to ensure the safety of both patients and them.

*It’s the third floor with no elevator and a spiral staircase*,* right? The patient weighs 140 kilos and has severe difficulty breathing. How are we supposed to manage this? (Int5/Part1)*

Despite these limitations, ACs describe how being able to provide relief for patients during transport is experienced as meaningful. Even within restricted environments, stabilizing or easing the patient’s respiratory distress and anxiety is viewed as an essential part of the caring act.

*What we can do in the apartment and during transport is a bit of a bonus. Sometimes the primary task is transporting the patient to the hospital. Being able to provide relief along the way is a plus. (Int5/Part2).*

#### Impact of supplies and equipment on patient care

ACs describe how the availability and quality of resources directly influence the emergency care they can provide to patients with breathlessness. They acknowledge that their ability to meet patients’ needs is limited by what is available in the ambulance, requiring them to adapt to the situation rather than strive for ideal conditions. This reality sometimes evokes feelings of constraint, but also acceptance of working within these practical boundaries.

*We also need to accept that we have only the resources we have. We don’t have access to all the possibilities and resources in the ambulance. (Int2/Part1).*

According to ACs, the quality and functionality of equipment play a crucial role in both patient assessment and treatment. They report that poor-quality tools, such as inadequate stethoscopes, conduct clinical assessments more difficult and may compromise decision-making, potentially affecting patient safety. In contrast, access to reliable and effective equipment enhances ACs’ confidence and their ability to provide safe care to patients.

*I believe we have excellent equipment in our vehicles for these situations. Feeling confident with the equipment is definitely an advantage. (Int7/Part1).*

ACs reflect on how technological developments and access to information may both support and complicate their clinical reasoning. For instance, having access to patients´ medical records during transport through digital tools can help tailor care based on previous conditions, but it could also bias clinical judgment and lead to assumptions that limit objective assessment. Similarly, the limited range of treatment options available in the ambulance can restrict what ACs can do for patients in severe distress, reinforcing the need for quick, prioritizing decisions.

*There are limited treatments we can provide for respiratory distress. It’s mostly either needle decompression if that’s indicated*,* but otherwise*,* it’s mainly inhalation and oxygen. We also have CPAP*,* if it’s a case of pulmonary edema. (Int3/Part2).*

### Navigating professional judgment within institutional boundaries

#### Balancing guidance and clinical judgment

ACs describe how they use institutional protocols and policies when caring for patients with breathlessness. They explain that these guidelines provide a basis for decision-making, particularly in acute care situations. According to ACs, the policies offer a sense of safety and structure, especially in high-stress scenarios. Adhering to protocols is described as helping to ensure consistency and accountability in clinical decisions.

*Yes*,* if you follow the guidelines*,* you’ve done your job.* (Int1/Part1).

However, ACs also express that policies can be restrictive when patients’ conditions fall outside standardized recommendations. In critical situations, such as severe respiratory or circulatory failure, strict adherence may limit available treatment options, leaving ACs feeling uneasy and powerless. The inability to administer certain interventions, such as airway support using continuous positive airway pressure (CPAP) due to contraindications, reinforces ACs’ sense of vulnerability when patients deteriorate despite appropriate efforts.

*CPAP has its limitations*,* as there are certain contraindications. For instance*,* you can’t use it if the patient has decreased consciousness. I feel uneasy when treatment options are restricted due to the patient’s physical state.* (Int1/Part2)

ACs highlight that drawing on their previous clinical experience and clinical reasoning is crucial for balancing guideline adherence with flexible decision-making. While policies serve as a framework, the ability to deviate from guidelines, when necessary, with supported communication with physicians, is described as part of their professional judgment. According to ACs, this adaptive approach is particularly evident in complex or pediatric cases that occur more seldom, where individualized assessment and planning beyond standard routines are required to meet patients’ specific needs.

*When it involves children*,* I check the child’s weight and consider factors like whether it might be croup or something else. This requires a different level of planning*,* including what equipment we need to bring to the patient. *(Int2/Part1)

### Accepting and adapting to limitations

ACs describe situations in which patients’ conditions exceed what can be treated in ambulance care. They emphasize that their role is to stabilize patients and ensure survival until hospital care is available, rather than attempting to resolve conditions beyond their scope. Accepting these limitations is highlighted as a crucial part of their professional practice.

*But they survived until they reached the hospital*,* or both of them survived. But… when the breathlessness is so severe… and there’s a bigger issue that you can’t resolve in an ambulance*,* you just try to manage the symptoms with what you have.* (Int5/Part2)

ACs describe feelings of frustration and inadequacy in the care of patients with breathlessness when available interventions reach their limits. Even after performing all possible measures, they encounter situations in which no further medical actions can be taken. Accepting these limitations is described by ACs as essential for maintaining a focus on patient safety and for providing satisfactory care within the resources available in the ambulance.

*Yes*,* but you reach a point where we’ve done all we can… we’ve secured the airway*,* we’ve set up the CPAP. There’s not much more we can do. Now*,* it’s almost up to the patient to pull through. What can be done once we reach the hospital*,* I don’t know*,* but at least we’ve taken the next step in the chain of care. Now*,* there’s nothing more I can do.* (Int6/Part1)

ACs stress that adapting to limitations requires prioritization, continuous assessment, and flexible decision-making. They describe patterns of practice and strategies such as closely monitoring patients, providing reassurance, and adjusting the pace of care according to patients’ responses and available interventions. Being present and maintaining composure are described as meaningful contributions to patient outcomes, even when further interventions are not possible at the emergency scene.

*No*,* we might not be able to do more medically*,* but we can still do more by just being there with the patient.* (Int5/Part2)

ACs underline that accepting limitations is not synonymous with inaction. Rather, accepting limitations involves using professional judgment, adaptation, and creativity within the constraints of ambulance care. Caring for patients with breathlessness within these boundaries is described as requiring resilience and a focus on achievable goals, ensuring that patients receive appropriate care until more advanced treatment becomes available.

*At the same time*,* I don’t really know what else we could do*,* considering we work in an ambulance. We must work within the limitations of our environment. In an emergency room*,* there are many other treatments available*,* but we don’t have those options. So*,* yes*,* it can be frustrating*,* but we must accept the limitations of our setting. I’m not sure what more we could do in such situations. *(Int8/Part2)

### Navigating responsibility in uncertain and distant care situations

#### Balancing uncertainty and responsibility in distant emergency care

ACs describe long transport distances as a major source of stress and emotional burden when caring for critically ill patients with breathlessness in ambulance care. Even when interventions are effective, the awareness that these patients are far from hospital support creates feelings of powerlessness and concern about what may happen during the journey. ACs emphasize the challenge of maintaining composure while continuing to provide care and reassurance under such conditions.

*But I work out in a smaller ambulance station*,* and when you’re out at rural area*,* it takes a long time to drive in*,* no matter how urgent it is. It takes a while*,* and you notice that*,* sure*,* you’re keeping them alive*,* but they’re not getting any better. And then… I feel a bit powerless*,* as if I can’t do more because I’ve already done everything I can.* (Int1/Part1)

ACs further describe that transporting critically ill patients with breathlessness over long distances demands constant vigilance and adaptation. They explain how they manage complex interventions, such as breathing support using CPAP, continuous monitoring of oxygen saturation, and handling patients’ declining consciousness alongside restlessness over extended periods. ACs emphasize that having a colleague in the back of the ambulance or access to additional resources is essential for maintaining patient safety. ACs form a collegial relationship of mutual dependence. Nevertheless, prolonged exposure to these high-stakes situations is described as contributing to stress and a sense of inadequacy.

*You start to think*,* ‘Why aren’t there more hospitals closer by?’ That feeling of inadequacy hits you. We keep driving*,* and he tolerates the CPAP enough to maintain an oxygen saturation of around 80–85%*,* but it’s still very critical. Eventually*,* as we’re on the road*,* I have a colleague with me in the back*,* so we’re two of us handling the situation.* (Int6/Part1)

ACs describe caring for patients with breathlessness as involving the provision of emergency treatments in situations where patients’ lives may be threatened. They experience that their working conditions in the pre-hospital setting are characterized by limited resources and distance to hospital care, which creates uncertainty regarding what can be safely managed during ambulance transport. Hospitals, with access to a wider range of treatment procedures and resources, are perceived as necessary for addressing more complex clinical needs. Within these conditions, ACs describe a constant balancing of professional responsibility, where decisions must be made under uncertainty about patient deterioration while managing the practical constraints of time, distance, and available resources.

*And especially with just a five-minute transport time to the hospital… hopefully they won’t deteriorate much either. It would be worse with several hours of transport. *(Int3/Part2)

#### Ambiguity in non-conveyance decisions

ACs describe facing ambiguous conditions when caring for patients with breathlessness in their homes. They explain that making non-conveyance decisions is therefore one of the most complex and emotionally challenging aspects of their work. ACs describe how expectations regarding when transport to hospital is considered necessary have changed over time, which has altered their clinical responsibilities within ambulance services.

*We now have different requirements. When I first started in ambulance services many years ago*,* we would transport everyone to the emergency department. Nowadays*,* we don’t transport everyone. we only take patients to the hospital if there is a valid reason to do so. (Int6/Part2)*

ACs express concern that non-conveyance decisions involving patients with breathlessness place substantial responsibility on them as clinicians. They describe how uncertainty and second thoughts often persist after such encounters. Some recount returning to patients they had initially decided not to convey or follow up with a phone call to ensure that the patient’s condition remains stable. These actions are described as ways of managing the emotional burden and professional responsibility associated with non-conveyance decisions.

*I think it’s quite common to worry about a patient you leave at home*,* wondering what happens next. One simple solution I’ve come up with is to call the patient two hours later to check them. Many people forget this*,* but it’s something you can actually do.* (Int7/Part1)

ACs also describe how guidelines and organizational expectations influence non-conveyance practices within ambulance services. They reflect on how discussions in the workplace increasingly emphasize decisions related to transport and care pathways. While ACs acknowledge the presence of such frameworks, they describe feeling pressure and vulnerability when balancing patient safety, professional judgment, and organizational demands.

*In such situations*,* one might feel more vulnerable. But now*,* we are working according to the ‘handover or optimize care level’ guidelines*,* and there is a lot of emphasis on these matters because they impact finances and budgeting.* (Int7/Part2)

## Discussions

This study provides important insights into ACs experiences of their physical, psychosocial and organizational working conditions when caring for patients with breathlessness, an area that has so far received limited attention in pre-hospital research. By addressing this gap, the study contributes to a deeper understanding of how the conditions of work under which ambulance care is delivered shape not only clinical decision-making but also the character and quality of the care provided. The main finding highlights that working conditions emerge as central factors that both enable and constrain ACs when caring for patients with breathlessness. This underscores that care cannot be understood solely as an individual or relational act, but as something fundamentally conditioned by the organizational, physical, and time-related working conditions in which it unfolds. Consequently, ACs’ caring practices need to be understood in close relation to the conditions under which they are carried out. Against this backdrop, one key finding was that ACs sometimes had to deprioritize their own safety to meet patients’ immediate clinical needs, such as maintaining a patent airway during transport. From a working conditions perspective, this illustrates a structural tension within ambulance services producing hazards and risks, where ACs are expected to deliver high-acuity care in environments that do not consistently support their own safety. Previous research has shown that ambulance personnel may be exposed to risks when they unfasten seat belts to restrain, calm, or provide care to patients who have loosened safety restraints [43, 44]. While such actions may be necessary in acute situations, they expose ACs to physical strain and increase the risk of injury, particularly when caring for patients with breathlessness [44, 45]. In line with these findings, the present study suggests that current ambulance work environments do not fully support safe practice, highlighting the need for organizational and structural interventions aimed at improving working conditions [43–45]. Ultimately, such conditions not only affect staff safety but also shape the circumstances under which patients receive care.

Within these constrained environments, ACs described how institutional guidelines function as an important source of structure and legitimacy in clinical decision-making when caring for patients with breathlessness. According to the participants, guidelines contribute to a sense of security in high-stress situations, yet they may also become restrictive when patient conditions fall outside standard recommendations or when interventions are contraindicated. These findings align with previous research showing that pre-hospital guidelines are often developed without sufficient input from end-users, are inconsistently operationalized, and may fail to capture the complexity of emergency care [13, 46, 47]. As a result, clinicians frequently rely on experience, intuition, and pattern recognition to tailor care to the individual patient, an approach consistently described in pre-hospital research [48–52]. The present study extends this understanding by illustrating how ACs actively develop adaptive strategies, such as modifying guidelines, consulting colleagues, or finding creative solutions, to manage situations where standard protocols are insufficient. Similar forms of adaptation have been described in studies of resilience in pre-hospital and emergency care, highlighting how clinicians navigate organizational, environmental, and clinical constraints while striving to maintain patient safety and care quality [53, 54]. From this perspective, strengthening guidelines to better address rare or complex scenarios, alongside structured collegial support, may help ACs balance professional judgment with organizational expectations. In addition, emerging decision-support tools, including machine learning–based systems, could potentially offer real-time guidance by integrating patient data, vital signs, and historical outcomes. Such tools may reduce decisional burden and support patient safety while complementing, rather than replacing, professional judgment [55]. However, such tools and guidelines may not eliminate uncertainty but instead redistribute responsibility for decision-making, leaving clinicians to interpret and apply recommendations in complex and time-sensitive situations.

At the same time, the findings indicate that ACs develop adaptive strategies to manage the inherent limitations of ambulance care, where stabilization rather than cure becomes an accepted and meaningful care goal. While accepting such limitations may reflect professional maturity, it may also, at least in part, represent an adaptation to organisational constraints that shape what is possible within the ambulance context [56]. This suggests that adaptation is not only an individual coping strategy, but also a response to structural conditions that continuously shape and constrain clinical work. Over time, such conditions may shift responsibility for managing system limitations onto individual clinicians. This sense of insufficiency appears to arise not only from clinical limitations but from a perceived inability to respond adequately to the patient’s expressed or unspoken needs in moments of profound vulnerability. When ACs are unable to offer the care they judge to be necessary, the ethical tension extends beyond professional standards to the relational responsibility inherent in the care encounter [17]. These adaptations appear to be a necessary part of everyday ambulance work and can be understood as expressions of resilience within a system characterized by persistent resource constraints and structural shortcomings [57, 58]. However, while flexibility and “making do” enable care delivery under strained conditions, they may also normalize improvisation and obscure systemic deficiencies, allowing structural problems to remain unaddressed [59, 60]. Moreover, these adaptive strategies may themselves constitute a significant ethical challenge. When ACs are repeatedly required to compromise, accept, or recalibrate aspects of care due to external constraints, this can give rise to moral stress and persistent feelings of insufficiency. Such experiences become particularly pronounced in situations where responsibility for patient safety is high while institutional support remains limited [57, 58]. This ethical tension is especially evident in the care of patients with breathlessness, a condition closely associated with fear, vulnerability, and uncertainty [18, 19], where discrepancies between required and achievable care are often acute. In this way, the findings highlight how working conditions in ambulance services are closely intertwined with ethical practice and care quality, underscoring the importance of addressing systemic conditions not only as a matter of occupational health, but also as a matter of patient safety [56, 61].

Finally, the present findings draw attention to non-conveyance decisions involving patients with breathlessness as particularly complex and burdensome for ACs. This complexity appears to be reinforced by a broader shift within ambulance services from routine conveyance toward more selective transport decisions, increasing reliance on individual clinical judgment in situations marked by uncertainty. Previous research similarly shows that limited availability of comprehensive non-conveyance guidelines places greater responsibility on clinicians’ experience and judgment, thereby increasing decisional pressure and perceived risk to patient safety [56, 62]. Factors such as clinicians’ confidence, educational background, and access to collegial or physician consultation further influence how responsibility is managed in these decisions [63]. Breathlessness represents a symptom with a potentially unstable trajectory, and non-conveyed patients with respiratory complaints have been shown to face an increased risk of adverse outcomes, including higher short-term mortality, particularly when abnormalities in vital signs are present [64, 65]. The concern expressed by ACs in the present study, manifested through follow-up phone calls or return visits, may be understood as attempts to bridge gaps in the formal care pathway, where responsibility for post–non-conveyance monitoring remains unclear. When such follow-up is initiated on an individual basis, outside formal structures, it constitutes additional, unrecognized work potential implications for clinician workload and wellbeing. This aligns with earlier findings showing that a substantial proportion of non-conveyed patients seek further care within a short time frame, suggesting challenges in ensuring safety once the ambulance encounter has ended [66]. This lack of formalised follow-up structures means that responsibility for patient monitoring becomes fragmented, relying on individual initiative rather than shared system-level processes. Notably, one participant in the present results explicitly linked non-conveyance decisions to budgetary and financial considerations, suggesting that resource management considerations may, in some contexts, influence clinical decision-making alongside patient-centred reasoning [67, 68]. From the patient’s perspective, non-conveyance may mark the end of the ambulance encounter but not the resolution of distress, uncertainty, or fear associated with breathlessness [18, 19]. Without such structures, there is a risk that non-conveyance functions less as a resolution of patient need and more as a redistribution of responsibility within the healthcare system, contributing to repeated care-seeking and prolonged uncertainty for both patients and ACs. This reflects a broader pattern in which organisational efficiency goals are translated into individual clinical encounters, thereby shifting system-level responsibility onto frontline clinicians [67–69]. Similar tensions have been described previously [56, 57, 69], where insufficient organizational support, limited feedback, and unclear care pathways lead clinicians to compensate through individual adaptations rather than shared responsibility. In this way, non-conveyance may function as a mechanism through which system-level priorities are enacted at the point of care, with implications for both patient safety and clinician burden.

## Strengths and limitations

This study has several strengths. The research team consisted of experienced pre-hospital researchers, including expertise in simulation research, which informed the study design and data collection. Two team members were RNs with specialist training in intensive care and ambulance care, bringing extensive clinical experience. The team also included a senior lecturer with expertise in management and organization within healthcare, as well as research experience in ambulance services, contributing additional insight into organizational and systemic aspects of pre-hospital care. High-fidelity simulation provided participants with a shared reference point prior to interviews, which was valuable given limited access to real-world pre-hospital settings. Dyadic interviews facilitated exploration of shared experiences, and the involvement of multiple interviewers strengthened credibility. Participants were recruited from different ambulance stations and varied in clinical experience, contributing to a diverse and nuanced understanding of care for patients with breathlessness. Although different analytic approaches were used, these were not parallel methods but separate analyses of the same dataset. In our previous publication, the focus was on ambulance clinicians’ experiences of caring for patients with breathlessness whereas the present study explores working conditions as an emergent aspect of the dataset, requiring a more interpretive analytic approach. The rationale for adopting a different analytic approach was to move beyond descriptive patterns and instead explore more latent and contextual dimensions of meaning that were not the primary focus of the initial analysis. This enabled a deeper and more nuanced understanding of the dataset from a different interpretive perspective. Reflexive thematic analysis [40] was applied to examine the interview data, enabling an in-depth exploration of participants’ perspectives while acknowledging the inherent subjectivity of qualitative interpretation. Collaborative coding and theme development provided opportunities for critical reflection and identification of potential blind spots, enhancing the richness and transparency of the findings. It should be noted, however, that the interpretations cannot claim to fully represent participants’ intentions, as qualitative analysis is inherently interpretive. Consensus was not sought, in line with the methodological emphasis on reflexivity rather than uniform agreement.

Some limitations should be noted. Snowball sampling may carry a risk of selection bias, but consecutive data collection and independent interviews minimize this risk. None of the interviewers had a background in ambulance care, which may have encouraged open descriptions of experiences. In some dyadic interviews, more experienced participants dominated, potentially limiting contributions from less experienced colleagues. Data were collected in 2021, and although some organizational changes may have occurred, the fundamental conditions of ambulance work, including high clinical acuity, time pressure, resource limitations, and reliance on individual clinical judgment, remain largely unchanged. Therefore, the findings are still relevant to current ambulance practice. The nurse-led structure of Swedish ambulance services, together with variation in specialist training, may limit transferability to paramedic-led systems internationally. Finally, the study was conducted at a limited number of ambulance stations in one region, but the findings still offer insights relevant to ambulance care and the management of patients with breathlessness across contexts.

## Conclusions

The findings demonstrate that working conditions within ambulance services with contingencies and constraints play a decisive role in shaping how care for patients with breathlessness is delivered, negotiated, and experienced. Caring for patients with breathlessness takes place in pre-hospital environments marked by clinical urgency, uncertainty, and limited time, information, resources, where ACs with mandated responsibilities are required to balance patient needs, personal safety, and organizational expectations that are not always aligned. Acceptance of limited resources restraining practice emerges as a necessary professional strategy that enables ACs to manage everyday work. At the same time, this acceptance may generate ethical, relational and organizational consequences, particularly when constraints limit the ability to respond to patients’ vulnerabilities, fears, and needs for security. These tensions are especially evident in non-conveyance decisions, where responsibility is often individualized and patient safety extends beyond the immediate ambulance encounter. By making visible how working conditions shape both clinical decision-making and the care encounter, the findings highlight that limited resources are not neutral constraints but integral to the care patients receive. Recognizing this relationship may support shared responsibility within ambulance services and help ensure that acceptance of limitations does not occur at the expense of patients’ experiences of safety, dignity, and care.

## Electronic Supplementary Material

Below is the link to the electronic supplementary material.

Supplementary Material 1

Supplementary Material 2

## Data Availability

The datasets used and analyzed during the current study are available from the corresponding author on reasonable request.

## Abbreviations

AC: Ambulance clinician
RN: Registered nurse
CPAP: Continuous positive airway pressure
