# Supplementary material for: “We need to accept our limited resources”: a qualitative study exploring ambulance clinicians’ experiences of working conditions when caring for patients with breathlessness
Source: BMC Emerg Med. 2026 May 28;26:159. doi: 10.1186/s12873-026-01620-w (PMC13227658; doi:10.1186/s12873-026-01620-w)
Supplement: Supplementary file 2 — Supplementary Material 2 [file 12873_2026_1620_MOESM2_ESM.docx]

| No | Interview Question |
| --- | --- |
| 1 | How did you experience caring for this patient who was struggling with her breathing? |
| 2 | What previous experiences do you have of caring for patients with breathlessness? |
| 3 | Do you think patients with breathlessness wait a long time before seeking help? |
| 4 | Can you recognize death anxiety in these patients? |
| 5 | How do you approach a patient who feels they are close to death? |
| 6 | What do you think a patient with breathlessness needs? |
| 7 | What do you think is the most important aspect of caring for a patient with breathlessness? |
| 8 | How do you usually calm a patient with breathlessness? |
| 9 | Is there anything else you would like to add that you think is important in caring for patients with breathlessness? |

Supplementary file 2. Overview of interview questions used in the study
